# Association between AST/ALT ratio and diabetic retinopathy risk in type 2 diabetes: a cross-sectional investigation

**DOI:** 10.3389/fendo.2024.1361707

**Published:** 2024-04-03

**Authors:** Xianhua Li, Wenqing Hao, Sen Lin, Nailong Yang

**Affiliations:** ^1^ Department of Endocrinology and Metabolism, The Affiliated Hospital of Qingdao University, Qingdao, China; ^2^ Department of Nursing and Hospital Infection Management, The Affiliated Hospital of Qingdao University, Qingdao, China; ^3^ Department of Endocrinology and Diabetes Department, Shouguang People’s Hospital, Weifang, Shandong, China

**Keywords:** aspartate aminotransferases, alanine transaminase, diabetic retinopathy, diabetes mellitus, type 2, correlation

## Abstract

**Objective:**

This study aimed to explore the association between the aspartate aminotransferase to alanine aminotransferase ratio (AST/ALT ratio) and diabetic retinopathy (DR) in patients with type 2 diabetes.

**Methods:**

In this cross-sectional study, clinical data from 3002 patients with type 2 diabetes admitted to the Department of Endocrinology of our hospital between January 1, 2021, and December 1, 2022, were retrospectively collected. Measurements of AST and ALT were conducted and diabetes-related complications were screened. The association between AST/ALT ratio and diabetic retinopathy was assessed using multivariate logistic regression, and a generalized additive model (GAM) was used to investigate nonlinear relationships. Subgroup analyses and interaction tests were also conducted.

**Results:**

Among the 3002 patients, 1590 (52.96%) were male and 1412 (47.04%) were female. The mean AST/ALT ratio was 0.98 ± 0.32, ranging from 0.37 (Min) to 2.17 (Max). Diabetic retinopathy was present in 40.47% of the patients. After multivariate adjustments, for each 0.1 unit increase in AST/ALT ratio, the risk of DR increased by 4% (OR = 1.04, 95% CI: 1.01–1.07, p=0.0053). Higher AST/ALT ratio quartiles were associated with Higher prevalence of DR (OR *vs*. Q1: Q4 = 1.34 (CI: 1.03–1.75, p=0.0303).The GAM and smoothed curve fit indicated a linear relationship between AST/ALT ratio and DR risk, with no significant interaction effects across different subgroups.

**Conclusion:**

Our study demonstrates a positive correlation between the AST/ALT ratio and diabetic retinopathy risk in type 2 diabetes, suggesting its potential role in assessing DR risk.

## 1 Introduction

Type 2 diabetes mellitus (T2DM) represents a significant global health challenge, affecting nearly half a billion people worldwide (1). Its prevalence is projected to increase dramatically, marking an escalating public health concern (2, 3). A prevalent microvascular complication of T2DM, diabetic retinopathy (DR), stands as a leading cause of blindness globally (4, 5). In China, the prevalence of DR among diabetic individuals exhibits considerable variation, with estimates ranging from 18.45% to 31.8%. This variation may reflect differences in study methodologies or demographic variations across regions, highlighting the complexity of managing DR within diverse populations (6, 7). Despite advancements in the understanding of DR, significant challenges in its early diagnosis and treatment remain (8, 9).

Liver enzymes, particularly Alanine aminotransferase(ALT) and Aspartate aminotransferase (AST), are emerging as significant markers (10, 11). The AST/ALT ratio, introduced by De Ritis in 1957, is a known indicator of liver function and has been associated with various systemic diseases, including cardiovascular, renal, and oncological conditions (12–15). Recent studies have also linked AST/ALT ratio with gestational diabetes and metabolic syndrome in children, expanding its relevance beyond liver diseases (16, 17). However, the relationship between AST/ALT ratio and DR in T2D patients remains under-explored.

Considering the roles of AST and ALT in metabolic and inflammatory processes (18–21), investigating their relationship with DR may unveil new proxies for early diagnosis and risk stratification. This is particularly crucial for patients with T2DM, where early detection and accurate risk assessment of DR are essential to prevent severe vision loss (22, 23). Moreover, given the predictive value of the AST/ALT ratio in other systemic diseases (15, 24, 25), exploring its potential association with DR could offer fresh insights into the complexity of T2DM complications and facilitate personalized patient management strategies.

This study aims to address this gap by examining the association between the AST/ALT ratio and DR in T2DM patients. By understanding this relationship, we seek to contribute to the early detection and risk stratification of DR, a crucial step towards improving patient outcomes in T2DM.

## 2 Methods

### 2.1 Research setting and population

This single-center, cross-sectional study was conducted at the Endocrinology Department of Qingdao University Affiliated Hospital, between January 1, 2021, and December 1, 2022. Inpatients aged 18 and above, diagnosed with Type 2 Diabetes Mellitus (T2DM) as per the American Diabetes Association’s 2021 guidelines, were included. Exclusion criteria were meticulously defined to mitigate potential confounding factors, including:

Presence of severe systemic or ocular conditions (e.g., uveitis, glaucoma) that could affect the study outcomes.
Recent ocular treatments or surgeries within the past three months.
Significant liver diseases or unexplained AST and ALT elevations exceeding 2.5 times the upper limit.
Use of medications known to affect liver function significantly.
Malignant neoplasms, due to their profound systemic health impact and potential confounding effect.

**Figure 1:**
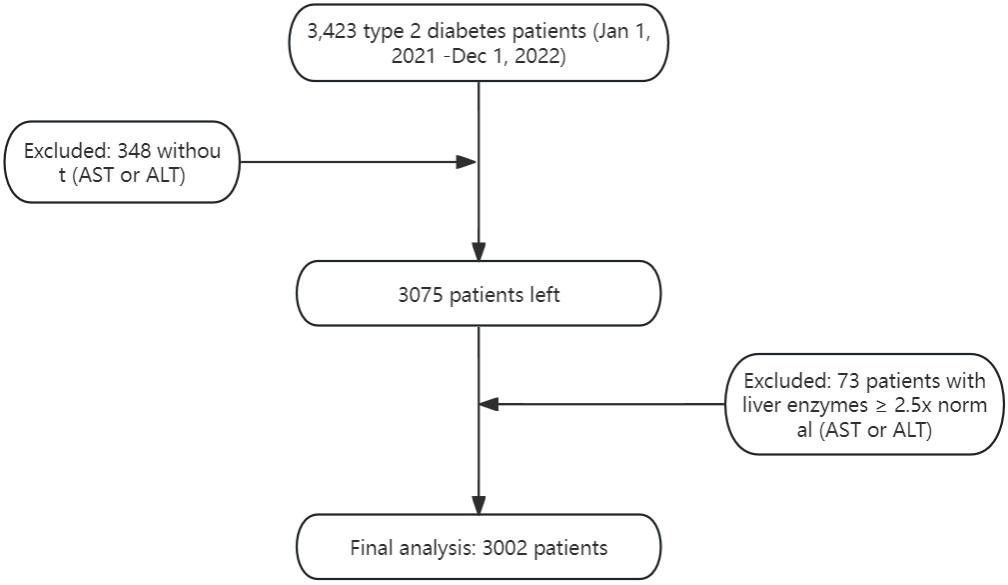
Flowchart of study population.

**Figure 1** illustrates the inclusion and exclusion criteria application.

### 2.2 Ethics statement

Adhering to the Declaration of Helsinki, informed consent was obtained from all participants. The study received ethical approval from the Ethics Committee of Qingdao University Affiliated Hospital (Approval No. QYFY WZLL 28254).

### 2.3 Measurement of AST and ALT

Blood samples were collected following an overnight fast to ensure accuracy. AST and ALT were quantified using the Hitachi 7600 analyzer, a method based on kinetic enzyme assay techniques. The established reference ranges were 15-40 U/L for AST and 9-50 U/L for ALT, aligning with clinical standards.

### 2.4 Diagnosis of diabetic retinopathy

The diagnosis of diabetic retinopathy (DR) in our study was rigorously conducted using a multi-modal imaging approach. Initial assessments involved slit lamp microscopy and optical coherence tomography to identify retinopathy changes during funduscopic examinations. This was followed by confirmatory diagnosis using 45° four-field stereoscopic digital photography with a Carl Zeiss Fundus Camera, which allowed for the detailed capture of images from both eyes across designated fields, eliminating the need for mydriasis (26).

Experienced ophthalmologists then evaluated these images, classifying DR according to the International Clinical Diabetic Retinopathy Disease Severity Scale into four severity levels. The identification of any severity lesion based on this scale was considered diagnostic of DR (26), ensuring an accurate and comprehensive diagnostic process (27).

### 2.5 Covariates

#### 2.5.1 Demographic and Clinical Data

Included variables were gender, age, diabetes duration, and complications.

#### 2.5.2 Anthropometrics

Measurements of height, weight, and body mass index (BMI) were conducted, with BMI calculated as weight in kilograms divided by height in meters squared. In aligning with specific regional considerations, we applied the WHO BMI Asian cutoffs to classify participants: <23 as underweight or normal, 23-25 as overweight, and >=25 as obese (28).

#### 2.5.3 Lifestyle Factors

Smoking and alcohol consumption were evaluated, with alcohol consumption defined as intake of 30 g or more weekly for over a year, and smoking defined by a history of 100 or more cigarettes smoked in one’s lifetime (29).

#### 2.5.4 Biochemical Analyses

Fasting blood samples enabled creatinine, fasting plasma glucose, liver function tests, HbA1c.All assessments, apart from HbA1c, utilized the Hitachi 7600 analyzer. Fatty liver diagnosis was ultrasound-based (30). Hypertension was identified through blood pressure measurements, following established criteria (31). The eGFR calculation employed the Andrew S. Levey formula (32), with diabetic nephropathy confirmed per KDIGO guidelines when urinary ACR ≥ 30 mg/mmol or eGFR < 60 mL/min/1.73m^^2^ (33). Diabetic Peripheral Neuropathy (DPN) Diagnosis: A comprehensive assessment integrated clinical, neurological examinations, and nerve conduction studies, adhering to recognized guidelines (34). DPN diagnosis utilized a structured approach: Clinically Evident DPN: Required at least two positive findings among sensory symptoms, signs, or reflex abnormalities consistent with distal symmetrical polyneuropathy. Abnormal Nerve Conduction Studies: Mandated at least one abnormal parameter (amplitude, latency, F-wave, or nerve conduction velocity) in two or more specific nerves.

### 2.6 Statistical methods

Continuous variables were reported as means and standard deviations if normally distributed, and medians with interquartile ranges (IQRs) for skewed distributions. Categorical variables were expressed as percentages. We utilized the Chi-square (χ^2) test for categorical variables, Student’s t-test for normally distributed continuous variables, and the Mann-Whitney U test for skewed continuous variables to evaluate differences in diabetic retinopathy status among participants. Additionally, differences among AST/ALT ratio quartiles were analyzed using the χ^2 test for categorical variables, One-Way ANOVA for normally distributed variables, and the Kruskal-Wallis H test for variables with skewed distributions.

The AST/ALT ratio was treated both as a continuous variable, assessed in increments of 0.1 units for a detailed examination of its relationship with diabetic retinopathy (DR) risk, and as a categorical variable through quartile conversion to facilitate group comparisons.

To identify variables potentially associated with diabetic retinopathy (DR), we initially conducted univariate logistic regression analysis. Following this, we employed logistic regression analysis, systematically adjusting for potential confounders across distinct models: Non-adjusted model: No adjustments were applied. Model I: Adjustments were made for essential demographic and clinical factors, including Age, Sex, and Body Mass Index (BMI).Model II: This model provided a comprehensive adjustment for an extended set of variables: Age, Sex, BMI, Duration of Diabetes, Fasting Blood Glucose (FBG), Glycated Hemoglobin (HbA1c), Diabetic Nephropathy (DN), and Diabetic Peripheral Neuropathy (DPN).

To assess non-linear relationships between the AST/ALT ratio and DR risk, Generalized Additive Models (GAM) were utilized. Additionally, subgroup analyses and interaction tests were conducted to investigate the effects of the AST/ALT ratio across different patient subgroups.

Statistical analyses were conducted using R software (The R Foundation) and EmpowerStats (http://www.empowerstats.com, X&Y Solutions, Inc., Boston, MA), ensuring the use of robust and reliable tools. Significance was determined by a two-sided P-value of less than 0.05, maintaining a standard criterion for statistical significance.

## 3 Results

In **Table 1**, our cross-sectional study included 3002 type 2 diabetes patients, divided into 1787 without diabetic retinopathy (Non-Diabetic Retinopathy group) and 1215 with the condition (Diabetic Retinopathy group). Key differences emerged in demographic and clinical measures.

**Table 1:** Baseline characteristics of participants.

	Non–Diabetic Retinopathy (n = 1787)	Diabetic Retinopathy (n = 1215)	P-value
Age (years old)	57.20 (13.11)	61.77 (10.94)	<0.001
Sex, *n* (%)			0.002
Male	988 (55.29%)	602 (49.55%)	
Female	799 (44.71%)	613 (50.45%)	
BMI (kg/m^2^)			0.002
<23	348 (19.58%)	290 (24.09%)	
23-25	398 (22.40%)	290 (24.09%)	
>=25	1031 (58.02%)	624 (51.83%)	
Smoking History, n (%)			0.093
Non-smokers	1282 (72.02%)	905 (74.79%)	
Smokers	498 (27.98%)	305 (25.21%)	
Alcohol Consumption History, n (%)			0.518
Non-drinkers	1326 (74.41%)	913 (75.45%)	
Drinkers	456 (25.59%)	297 (24.55%)	
Diabetic duration (years)			<0.001
<5	795 (44.49%)	250 (20.58%)	
5-10	409 (22.89%)	240 (19.75%)	
>=10	583 (32.62%)	725 (59.67%)	
FBG (mmol/L)	7.37 (2.29)	7.50 (2.84)	0.165
HbA1c (%)	8.46 (2.07)	8.77 (1.94)	<0.001
AST/ALT	0.94 (0.31)	1.03 (0.33)	<0.001
eGFR (mL/min per 1.73 m^2^)	96.38 (41.94)	94.95 (45.38)	0.383
Hypertension (%)			<0.001
No	858 (48.01%)	507 (41.73%)	
Yes	929 (51.99%)	708 (58.27%)	
Fatty Liver Disease			<0.001
No	835(46.73%)	745(61.32%)	
Yes	952(53.27%)	470(38.68%)	
Diabetic nephropathy			<0.001
No	1363 (76.27%)	750 (61.73%)	
Yes	424 (23.73%)	465 (38.27%)	
Diabetic peripheral neuropathy			<0.001
No	761 (42.59%)	239 (19.67%)	
Yes	1026 (57.41%)	976 (80.33%)	

Table Results Format: (N) Mean(SD).

FBG, Fasting Blood Glucose; HbA1c, Hemoglobin A1c; AST/ALT, aspartate aminotransferase to alanine aminotransferase; eGFR, Estimated Glomerular Filtration Rate.

Significantly, the Diabetic Retinopathy group was older and had a higher proportion of females. The duration of diabetes was notably longer in this group.

Clinically, the Diabetic Retinopathy group showed higher levels of Glycated Hemoglobin (HbA1c) and a significant difference in the AST/ALT ratio, reflecting a potential link to retinopathy severity. Prevalences of hypertension and diabetic nephropathy were also higher in the Diabetic Retinopathy group, underscoring the comorbidity burden in these patients. In **Supplementary Material 1**, we present the baseline characteristics of patients with Type 2 Diabetes Mellitus (T2DM), stratified by quartiles of the AST/ALT ratio.

In our preliminary univariate logistic regression analysis(**Table 2**), we observed a significant correlation between the AST/ALT ratio and the risk of diabetic retinopathy (DR) in patients with Type 2 Diabetes Mellitus (T2DM). Notably, the AST/ALT ratio, both as a continuous variable (OR: 1.98, 95% CI: 1.61 – 2.42, p < 0.0001) and when categorized into quartiles, showed a strong, positive association with DR risk, escalating across quartiles (Q1 as reference: Q2 OR: 1.55, Q3 OR: 1.64, Q4 OR: 2.16; all p < 0.0001). Other factors, such as age, female sex, longer diabetes duration, higher HbA1c levels, hypertension, fatty liver disease, diabetic nephropathy, and diabetic peripheral neuropathy, were also significantly associated with increased DR risk.

**Table 2:** Univariate logistic regression analysis: factors correlated with diabetic retinopathy.

	Diabetic Retinopathy
Age (years old)	1.03 (1.03, 1.04) <0.0001
Sex, *n* (%)	
Male	1.0(Ref)
Female	1.26 (1.09, 1.46) 0.0020
BMI categorical	
<23	1.0(Ref)
23-25	0.87 (0.70, 1.09) 0.2258
>=25	0.73 (0.60, 0.87) 0.0007
Smoking History, n (%)	
Non-smokers	1.0(Ref)
Smokers	0.87 (0.73, 1.02) 0.0935
Alcohol Consumption History, n (%)	
Non-drinkers	1.0(Ref)
Drinkers	0.95 (0.80, 1.12) 0.5185
Diabetic duration (years)	
<5	1.0(Ref)
5-10	1.87 (1.51, 2.31) <0.0001
>=10	3.95 (3.31, 4.73) <0.0001
HbA1c (%)	1.08 (1.04, 1.12) 0.0001
FBG (mmol/L)	1.02 (0.99, 1.05) 0.1654
eGFR (mL/min per 1.73 m^2^)	1.00 (1.00, 1.00) 0.3830
AST/ALT (per 1 change)	1.98 (1.61, 2.42) <0.0001
AST/ALT quartile	
Q1	1.0(Ref)
Q2	1.55 (1.25, 1.92) <0.0001
Q3	1.64 (1.32, 2.03) <0.0001
Q4	2.16 (1.75, 2.67) <0.0001
Hypertension	
No	1.0(Ref)
Yes	1.29 (1.11, 1.49) 0.0007
Fatty Liver Disease	
No	1.0(Ref)
Yes	0.55 (0.48, 0.64) <0.0001
Diabetic nephropathy	
No	1.0(Ref)
Yes	1.99 (1.70, 2.34) <0.0001
Diabetic peripheral neuropathy	
No	1.0(Ref)
Yes	3.03 (2.56, 3.59) <0.0001

OR (95%CI) Pvalue.

In our logistic regression analysis, as detailed in **Table 3**, we systematically adjusted for potential confounders to evaluate the association between the AST/ALT ratio and the risk of diabetic retinopathy (DR) across several models. Analyzing the AST/ALT ratio as a continuous variable, the unadjusted model revealed a significant association (OR: 1.09, 95% CI: 1.07 - 1.12, p < 0.0001). The association’s strength was slightly attenuated upon adjusting for age, sex, and BMI in Model I (OR: 1.05, 95% CI: 1.03 - 1.08, p < 0.0001). Further adjustments in Model II, incorporating variables such as duration of diabetes, fasting blood glucose, HbA1c, diabetic nephropathy, and diabetic peripheral neuropathy, continued to show a positive association, albeit reduced (OR: 1.04, 95% CI: 1.01 - 1.07, p = 0.0053). These findings suggest that the AST/ALT ratio was consistently associated with higher odds of DR in type 2 diabetes, even after accounting for various confounding factors.

**Table 3:** Relationship between AST/ALT and DR in different models.

Variable	Non-adjusted OR (95%CI) Pvalue	Adjust I OR (95%CI) Pvalue	Adjust II OR (95%CI) Pvalue
AST/ALT(per 0.1 change)	1.09 (1.07, 1.12) <0.0001	1.05(1.03, 1.08) <0.0001	1.04 (1.01, 1.07) 0.0053
AST/ALT subgroups			
Q1	1.0(Ref)	1.0(Ref)	1.0(Ref)
Q2	1.55(1.25,1.92) <0.0001	1.34(1.08,1.68) 0.0090	1.19(0.92,1.55) 0.1781
Q3	1.64(1.32,2.03) <0.0001	1.28(1.02,1.60) 0.0340	1.09(0.84,1.42) 0.4999
Q4	2.16(1.75,2.67) <0.0001	1.55(1.23,1.95) 0.0002	1.34(1.03,1.75) 0.0303

Non-adjusted model adjust for: None.

Adjust I model adjust for: Age; Sex; BMI.

Adjust II model adjust for: Age; Sex; BMI; Diabetic duration; FBG; HbA1c; DN; DPN.

When analyzing the AST/ALT ratio as a variable categorized into quartiles, we observed that higher AST/ALT ratio quartiles were associated with an increased prevalence of DR in Model II, illustrating a gradient of risk across quartiles (OR *vs*. Q1: Q2 = 1.19, 95% CI: 0.92–1.55; Q3 = 1.09, 95% CI: 0.84–1.42; and Q4 = 1.34, 95% CI: 1.03–1.75).

**Figure 2:**
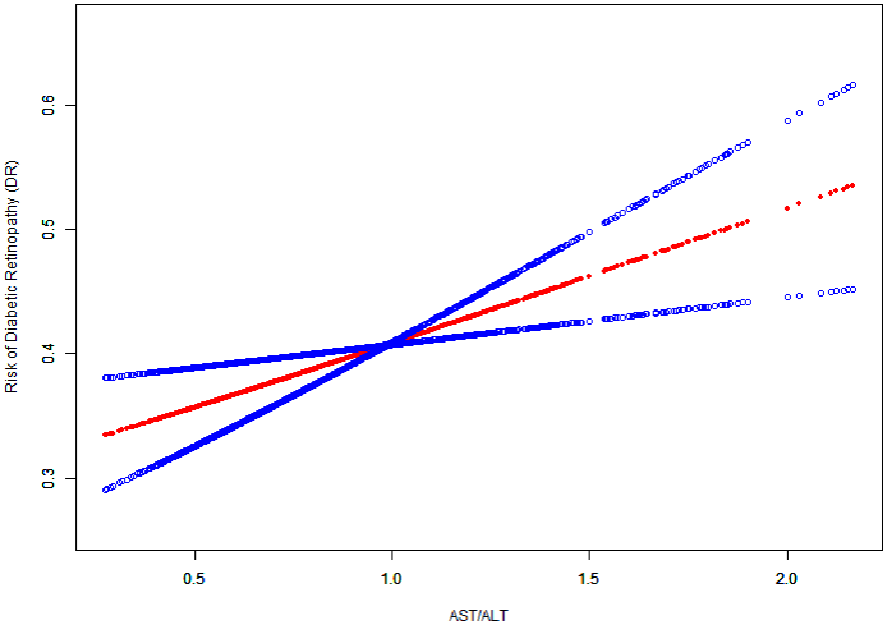
The Linear Relationship Between the AST/ALT Ratio and the Risk of Diabetic Retinopathy. Image Annotation: The generalized additive model plot delineates the association between AST/ALT Ratio (X-axis) and Risk of Diabetic Retinopathy (DR) (Y-axis).

In our study, we utilized generalized additive models (GAM) to explore the potential non-linear relationship between the AST/ALT ratio and diabetic retinopathy (DR), given the continuous nature of the AST/ALT ratio as depicted in **Figure 2**. Our analysis, however, identified a linear relationship between the AST/ALT ratio and DR risk. This finding was determined after comprehensive adjustments for a range of critical variables, including age, sex, body mass index (BMI), duration of diabetes, fasting blood glucose (FBG), Hemoglobin A1c (HbA1c), diabetic nephropathy (DN), and diabetic peripheral neuropathy (DPN).

**Figure 3:**
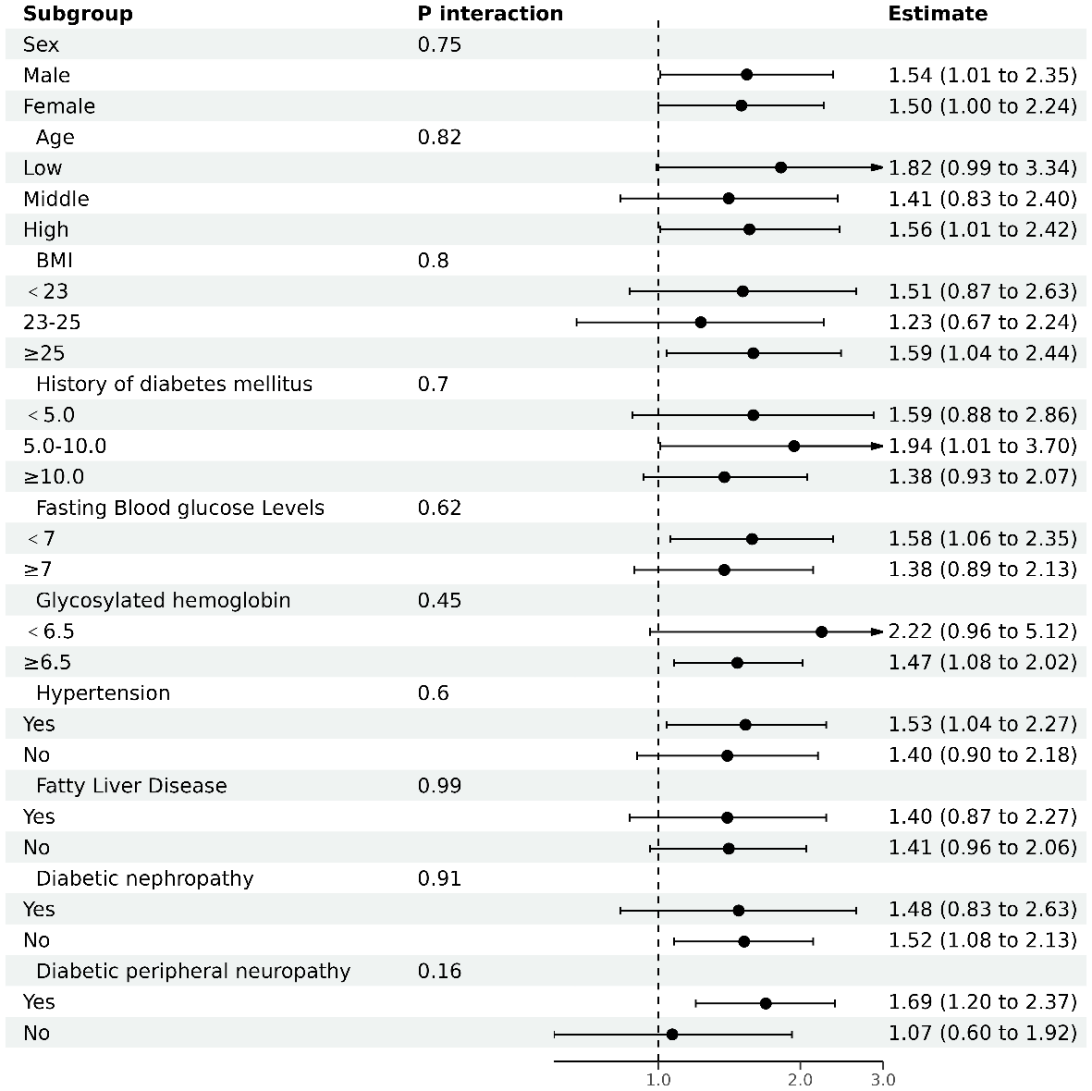
The results of subgroup analyses. Analysis adjusted for age, sex, body mass index (BMI), duration of diabetes, fasting blood glucose (FBG), Hemoglobin A1c (HbA1c), diabetic nephropathy (DN), and diabetic peripheral neuropathy (DPN), excluding the variable specific to each subgroup under investigation.

In our investigation, depicted in **Figure 3**, we performed stratified analyses to examine the association between the AST/ALT ratio and diabetic retinopathy (DR) risk across diverse subgroups. These subgroups were defined by several key characteristics. The results, illustrated in a forest plot, indicated a consistent association between the AST/ALT ratio and diabetic retinopathy across our subgroups. Notably, this association was evaluated based on a per 1 unit increase in the AST/ALT ratio. Despite variations in demographic and clinical characteristics, the interaction p-values all exceeded 0.05, suggesting a stable and robust relationship between AST/ALT ratio and diabetic retinopathy risk that is not significantly influenced by these stratifying factors.

## 4 Discussion

Our study’s primary findings indicate a positive correlation between the AST/ALT ratio and the risk of diabetic retinopathy (DR) in patients with type 2 diabetes. This suggests that patients with higher AST/ALT ratios should be promptly screened for diabetic retinopathy, aiding in the improved management of type 2 diabetic retinopathy. These results provide novel insight into the relationship between liver enzymes and diabetic complications, highlighting the importance of the AST/ALT ratio in the clinical assessment of diabetes patients.

The pathophysiology of DR encompasses oxidative stress, chronic inflammation, and altered metabolic pathways. Intriguingly, the AST/ALT ratio, indicative of changes in liver enzyme levels, is associated with these same pathophysiological factors, including oxidative stress, systemic inflammation, and insulin resistance (35, 36). This association suggests that altered AST/ALT ratios could play a role in the progression of DR.

Further, our findings are in line with other research that highlights the significance of AST/ALT ratios in various diabetic complications like peripheral neuropathy and nephropathy. These studies underscore the systemic impact of altered liver enzyme levels in diabetes (35, 37).

A notable strength of our study is the use of GAM, which effectively demonstrates the linear relationship between AST/ALT ratios and DR. Through rigorous statistical adjustments and consistent findings across different subgroups, our study suggests that each 0.1 unit increase in the AST/ALT ratio is associated with a 4% increase in the risk of DR.

Additionally, our comprehensive search of the PubMed database did not reveal any similar studies, highlighting the originality of our research. This study preliminarily suggests a potential association between the AST/ALT ratio and diabetic retinopathy (DR), thereby offering a new dimension in understanding possible biomarkers for diabetes and its complications.

However, this study still has some limitations. Being a cross-sectional analysis, it offers associative insights but cannot confirm causality (38). Additionally, the applicability of our results may be restricted to the Chinese population and might not extend to those with AST/ALT ratios beyond the 0.37 to 2.17 range.

In conclusion, our research sheds light on the AST/ALT ratio as a potential indicator associated with the risk of diabetic retinopathy in type 2 diabetes patients. These initial findings suggest that the AST/ALT ratio could be further investigated as a biomarker for assessing DR risk. Such investigations are important to validate these findings and explore the underlying biological mechanisms in more diverse populations and through prospective studies.

## 5 Conclusion

This study reveals a positive correlation between the AST/ALT ratio and the risk of diabetic retinopathy in type 2 diabetes patients. Further studies are essential to confirm these findings and investigate the underlying mechanisms across diverse populations.

## Data availability statement

The raw data supporting the conclusions of this article will be made available by the authors, without undue reservation.

## Ethics statement

This research was conducted in accordance with the principles set forth in the Declaration of Helsinki. Informed consent was obtained from all participants. The study received the endorsement of the Ethics Committee of the Affiliated Hospital of Qingdao University (Approval No. QYFY WZLL 28254).

## Author contributions

XL: Data curation, Formal analysis, Writing – original draft. WH: Data curation, Supervision, Writing – review & editing. SL: Data curation, Formal analysis, Writing – review & editing. NY: Supervision, Writing – review & editing.
