# Supplementary material for: Association between AST/ALT ratio and diabetic retinopathy risk in type 2 diabetes: a cross-sectional investigation
Source: Front Endocrinol (Lausanne). 2024 Apr 3;15:1361707. doi: 10.3389/fendo.2024.1361707 (PMC11021722; doi:10.3389/fendo.2024.1361707)
Supplement: Supplementary file 1 [file Table_1.docx]

Supplementary Material 1:Baseline characteristics of participants by the AST/ALT quartile.

| AST/ALT quartile | Q1(0.37,0.74) | Q2(0.74,0.93) | Q3(0.93,1.16) | Q4(1.16,2.17) | P-value |
| --- | --- | --- | --- | --- | --- |
| N | 707 | 767 | 772 | 756 |  |
| Age (years old) | 52.30 ± 13.49 | 58.10 ± 11.24 | 61.33 ± 10.78 | 63.99 ± 11.33 | <0.001 |
| Sex, *n* (%) |  |  |  |  | <0.001 |
| Male | 492 (69.59%) | 438 (57.11%) | 361 (46.76%) | 299 (39.55%) |  |
| Female | 215 (30.41%) | 329 (42.89%) | 411 (53.24%) | 457 (60.45%) |  |
| BMI (kg/m^2^) |  |  |  |  | <0.001 |
| <23 | 88 (12.54%) | 130 (17.02%) | 195 (25.46%) | 225 (30.04%) |  |
| >=23, <25 | 131 (18.66%) | 180 (23.56%) | 186 (24.28%) | 191 (25.50%) |  |
| >=25 | 483 (68.80%) | 454 (59.42%) | 385 (50.26%) | 333 (44.46%) |  |
| Smoking History, n (%) |  |  |  |  | <0.001 |
| Non-smokers | 447 (63.40%) | 548 (71.82%) | 589 (76.49%) | 603 (80.19%) |  |
| Smokers | 258 (36.60%) | 215 (28.18%) | 181 (23.51%) | 149 (19.81%) |  |
| AlcoholConsumption History, n (%) |  |  |  |  | <0.001 |
| Non-drinkers | 463 (65.58%) | 552 (72.16%) | 600 (77.92%) | 624 (83.09%) |  |
| Drinkers | 243 (34.42%) | 213 (27.84%) | 170 (22.08%) | 127 (16.91%) |  |
| Diabetic duration (years) |  |  |  |  | <0.001 |
| <5 | 357 (50.50%) | 267 (34.81%) | 229 (29.66%) | 192 (25.40%) |  |
| 5-10 | 146 (20.65%) | 193 (25.16%) | 172 (22.28%) | 138 (18.25%) |  |
| >=10 | 204 (28.85%) | 307 (40.03%) | 371 (48.06%) | 426 (56.35%) |  |
| FBG(mmol/L) | 8.06 ± 2.75 | 7.64 ± 2.39 | 7.19 ± 2.55 | 6.87 ± 2.25 | <0.001 |
| HBA1C(%) | 8.74 ± 1.91 | 8.76 ± 2.05 | 8.53 ± 1.98 | 8.35 ± 2.10 | <0.001 |
| AST/ALT | 0.61 ± 0.09 | 0.83 ± 0.05 | 1.03 ± 0.07 | 1.42 ± 0.22 | <0.001 |
| eGFR(mL/min per 1.73 m^2^) | 101.42 ± 43.36 | 96.58 ± 43.20 | 92.47 ± 42.58 | 93.31 ± 43.86 | <0.001 |
| Hypertension (%) |  |  |  |  | 0.017 |
| No | 356 (50.35%) | 342 (44.59%) | 347 (44.95%) | 320 (42.33%) |  |
| Yes | 351 (49.65%) | 425 (55.41%) | 425 (55.05%) | 436 (57.67%) |  |
| Fatty Liver Disease(%) |  |  |  |  | <0.001 |
| No | 232 (32.81%) | 370 (48.24%) | 453 (58.68%) | 525 (69.44%) |  |
| Yes | 475 (67.19%) | 397 (51.76%) | 319 (41.32%) | 231 (30.56%) |  |
| Diabetic nephropathy(%) |  |  |  |  | 0.816 |
| No | 500 (70.72%) | 546 (71.19%) | 545 (70.60%) | 522 (69.05%) |  |
| Yes | 207 (29.28%) | 221 (28.81%) | 227 (29.40%) | 234 (30.95%) |  |
| Diabetic peripheral neuropathy(%) |  |  |  |  | <0.001 |
| No | 297 (42.01%) | 266 (34.68%) | 233 (30.18%) | 204 (26.98%) |  |
| Yes | 410 (57.99%) | 501 (65.32%) | 539 (69.82%) | 552 (73.02%) |  |
| Diabetic Retinopathy(%) |  |  |  |  | <0.001 |
| No | 489 (69.17%) | 459 (59.84%) | 451 (58.42%) | 388 (51.32%) |  |
| Yes | 218 (30.83%) | 308 (40.16%) | 321 (41.58%) | 368 (48.68%) |  |

Note:

Table Results Format: (N) Mean(SD)
